# Supplementary figures and images for: Massive entry of BK Polyomavirus induces transient cytoplasmic vacuolization of human renal proximal tubule epithelial cells
Source: PLoS Pathog. 2024 Nov 21;20(11):e1012681. doi: 10.1371/journal.ppat.1012681 (PMC11581322; doi:10.1371/journal.ppat.1012681)

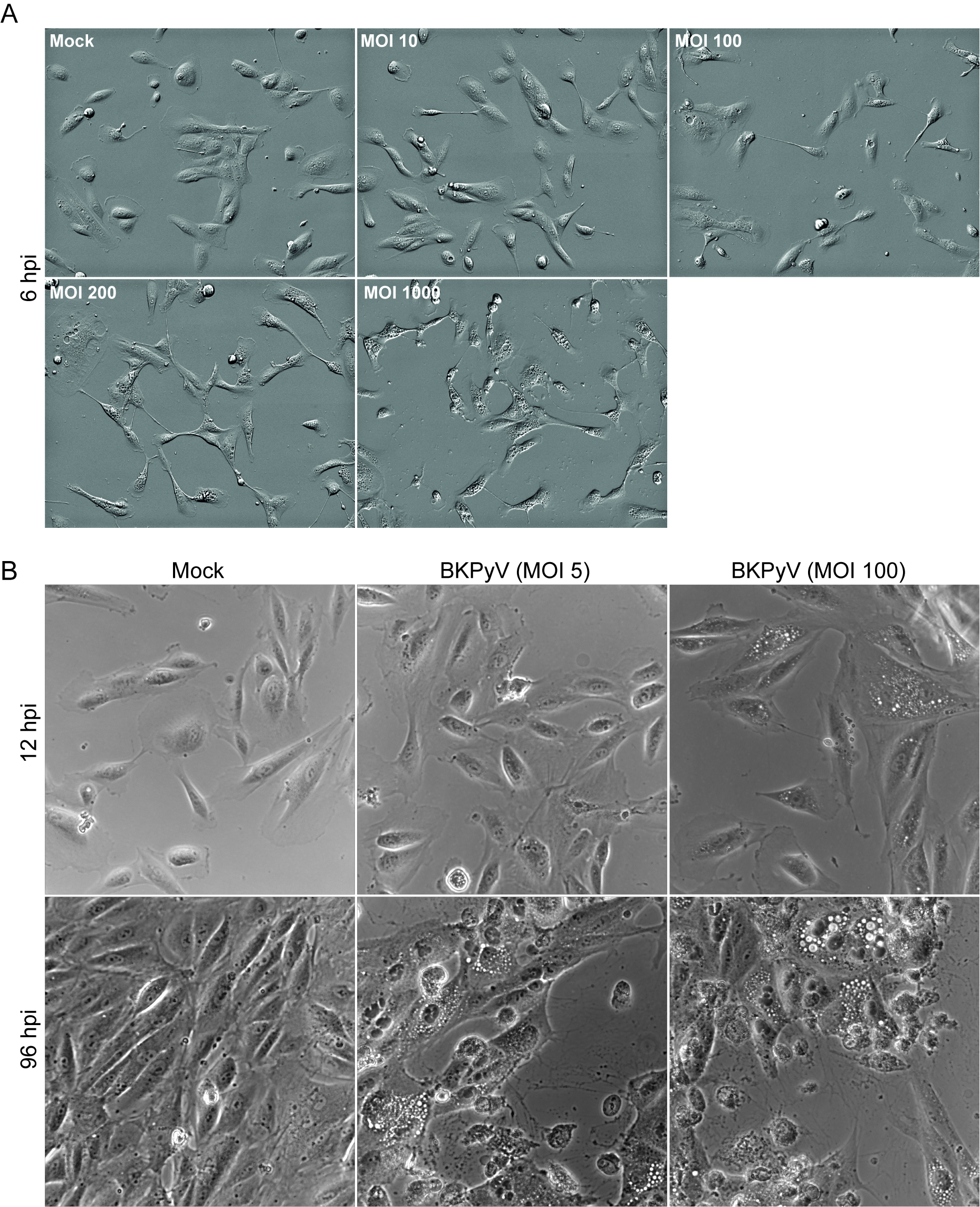

Supplement: S1 Fig — (A) Widefield microscopy with oblique contrast of BKPyV-infected RPTECs at 6 hpi (MOI 0.1, 1.0, 10, 200 and 1000). Images are representative images from Fig 1C. (B) Widefield microscopy of RPTECs from Sciencell infected with BKPyV (MOI 5 or 100) at 12 and 96 hpi. Representative images from a single experiment are shown. (TIF) [file ppat.1012681.s001.tif]

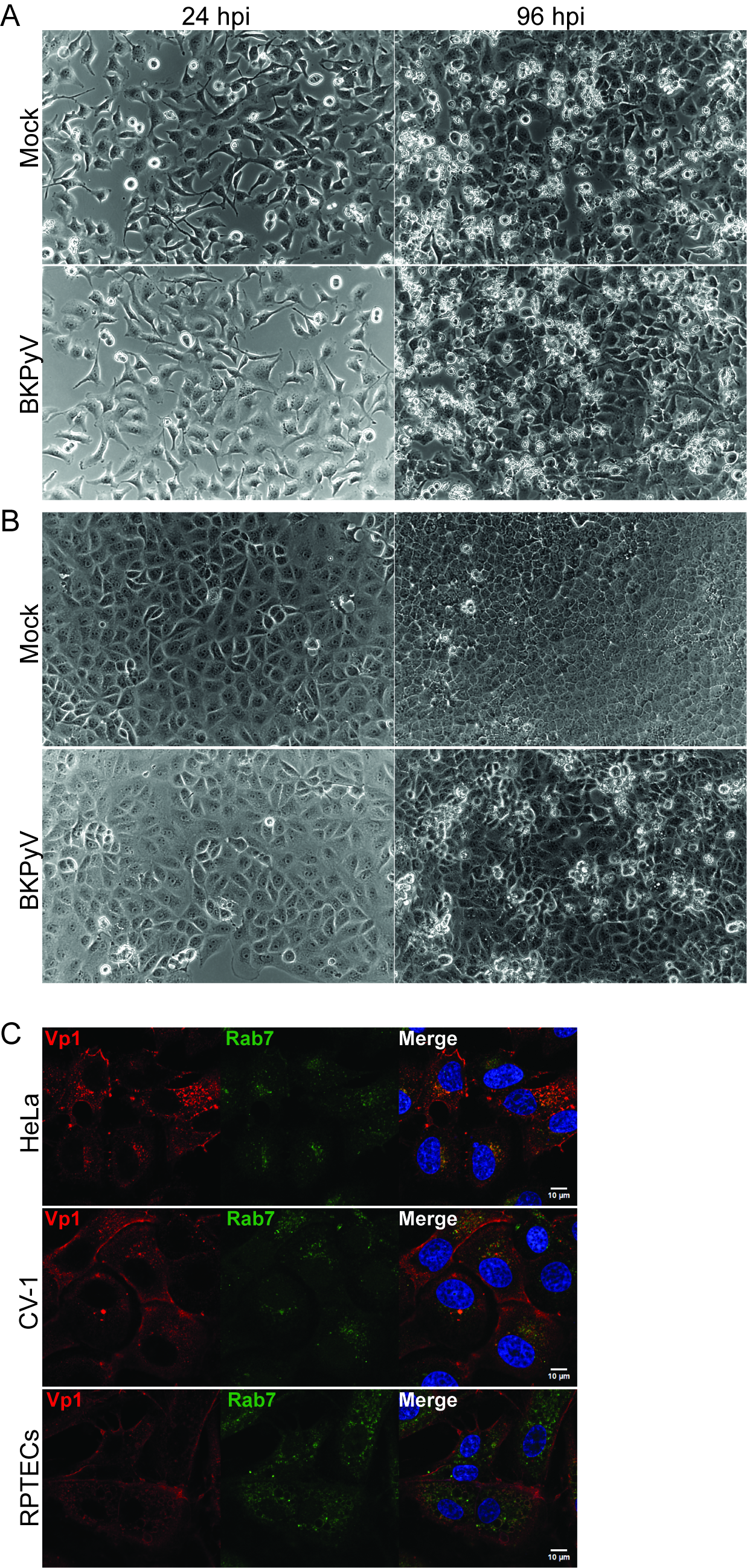

Supplement: S2 Fig — Phase-contrast images of Mock-infected and BKPyV-infected cells (MOI 100) at 24 hpi and 96 hpi, (A) HeLa cells, and (B) CV-1 cells. Representative images from three independent experiments are shown. (C) Confocal microscopy following immunofluorescence staining at 6 hpi of BKPyV-infected HeLa cells, CV-1 cells, and RPTECs (MOI 200), respectively. A mouse monoclonal antibody against Rab7 (red) was combined with a mouse monoclonal antibody against BKPyV Vp1 (green). Nuclei are stained with Dapi (blue). Representative images from a single independent experiment are shown. Scale bar = 10 μm. (TIF) [file ppat.1012681.s002.tif]

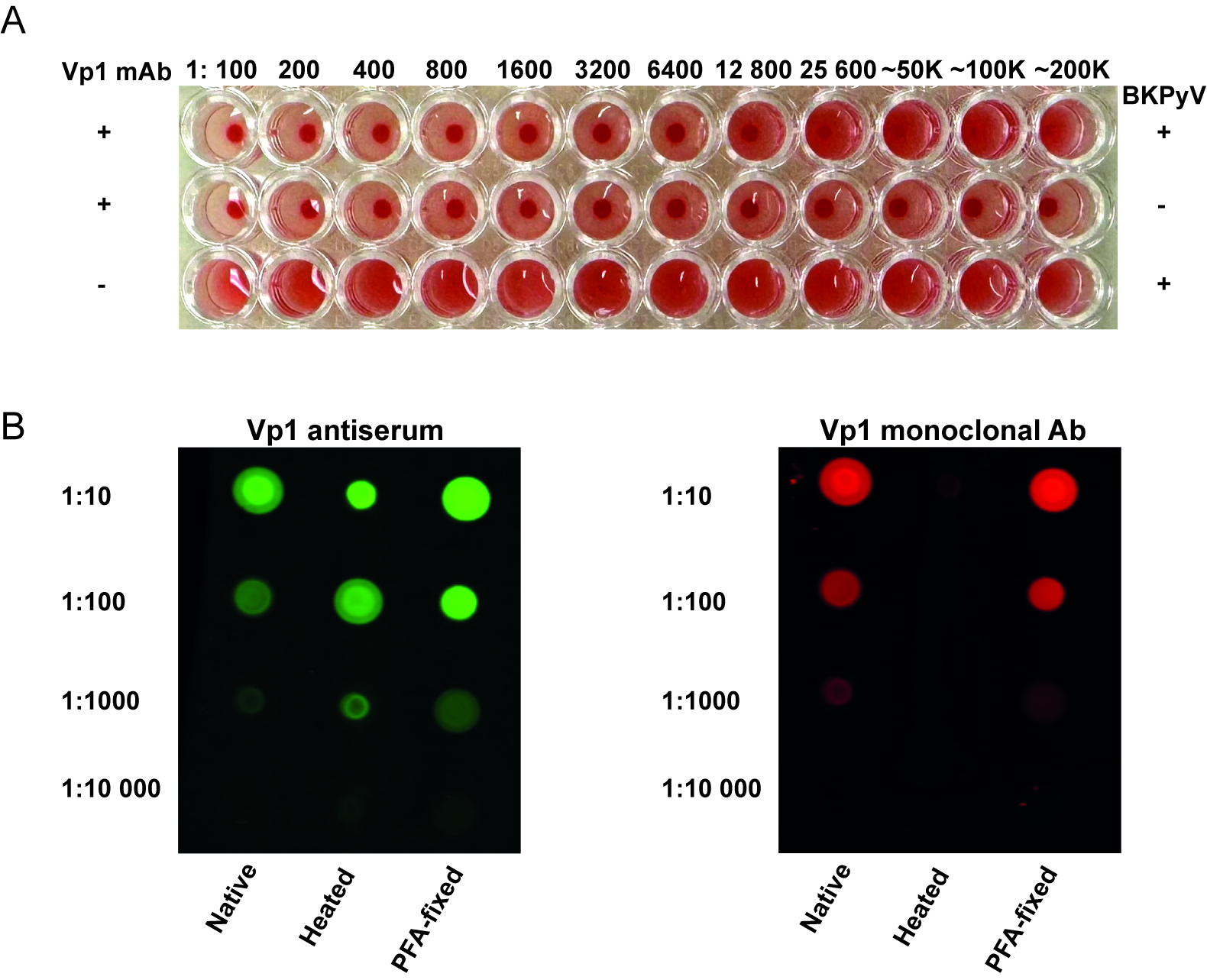

Supplement: S3 Fig — (A) Hemagglutination inhibition assay with BKPyV after pre-incubation with a mouse monoclonal antibody against BKPyV Vp1 (Vp1 mAb, Virostat 4942). A negative control without BKPyV and a positive control without the Vp1 mAb are included. Hemagglutination was fully inhibited with a 100–6400 times dilution and partly inhibited with a 12 800–51 200 times dilution. A representative image from two independent experiments is shown. (B) Dot blot of native (untreated), heat-treated, and PFA-fixed gradient-purified BKPyV. After treatment, 1 μl virus was applied to a nitrocellulose membrane before the membrane was blocked, stained with primary and secondary antibodies, and imaged with a LI-COR Imaging system. As primary antibodies, a rabbit BKPyV Vp1 antiserum and the mouse monoclonal antibody against BKPyV Vp1 (Virostat 4942) were used, respectively. (TIF) [file ppat.1012681.s003.tif]

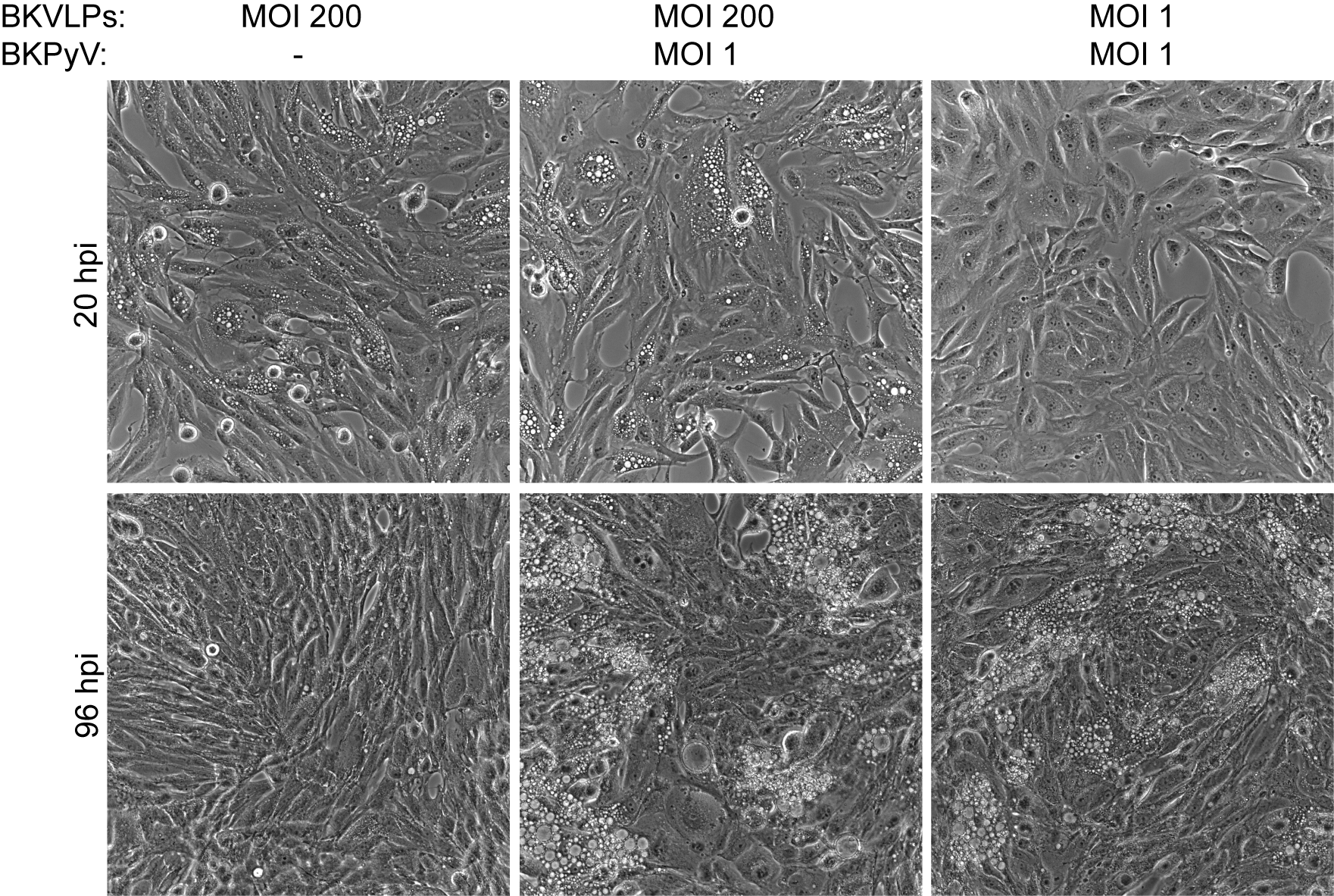

Supplement: S4 Fig — Phase-contrast images of RPTECs at 24 hpi and 96 hpi with only BKVLPs (equal to MOI 200 based on the hemagglutination titer), a combination of BKVLPs (equal to MOI 200) and BKPyV (MOI 1), or a combination of BKVLPs (equal to MOI 1) and BKPyV (MOI 1). Representative images from two independent experiments are shown. (TIF) [file ppat.1012681.s004.tif]

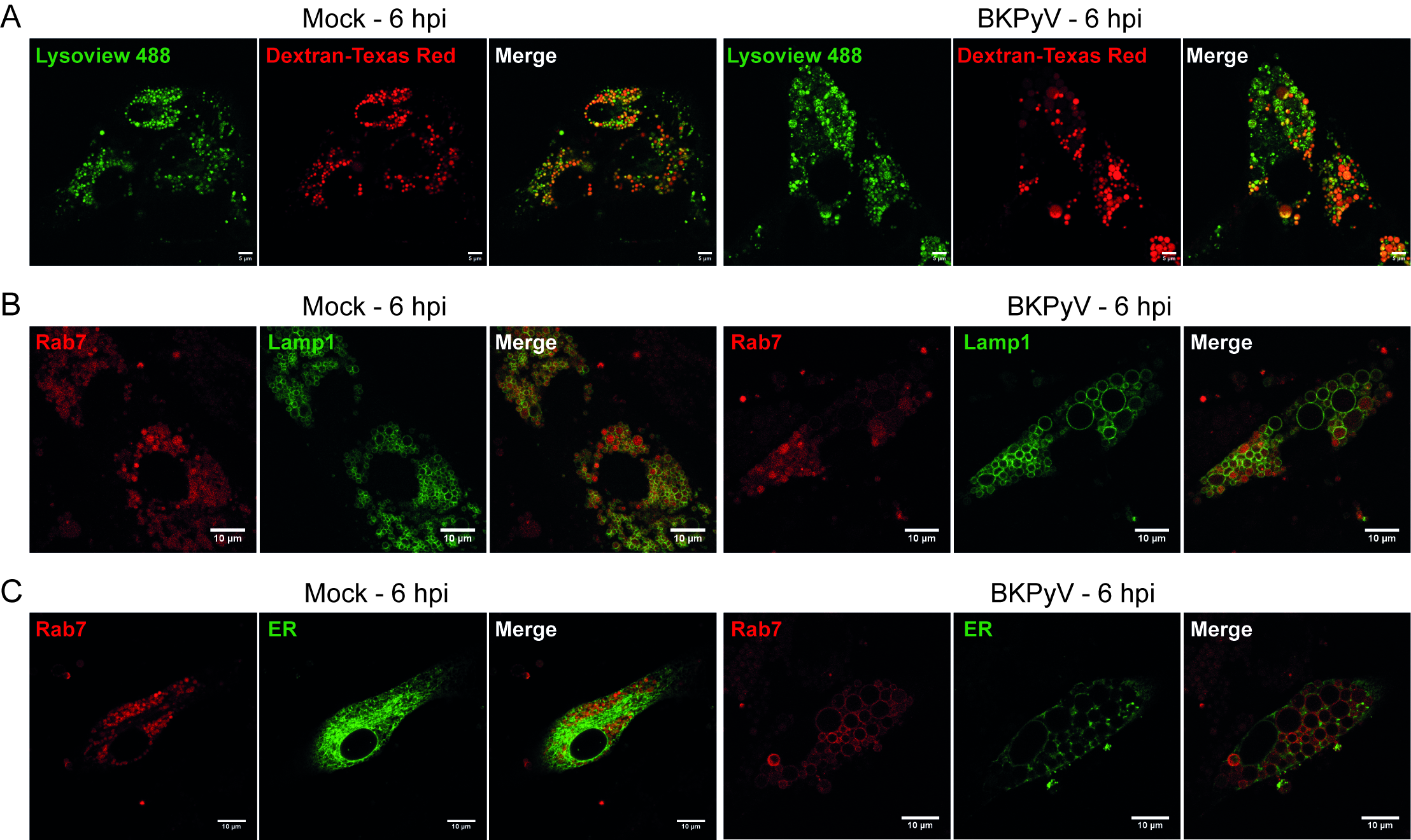

Supplement: S5 Fig — Live-cell confocal microscopy of mock-infected and BKPyV-infected RPTECs (MOI 100–200) with markers of endo-/lysosomes and the ER at 6 hpi. (A) RPTECs were incubated overnight with Texas Red conjugated dextran (red) and stained with Lysoview 488 (green). Scale bar = 5 μm. (B) RPTECs that transiently express Rab7-mCherry (red), a marker of late endosomes and lysosomes, and Lamp1 (green), a marker of lysosomes. Scale bar = 10 μm. (C) RPTECs that transiently express Rab7-mCherry (red) and mTurquoise2-ER (green). Scale bar = 10 μm. Representative images from two independent experiments are shown. (TIF) [file ppat.1012681.s005.tif]

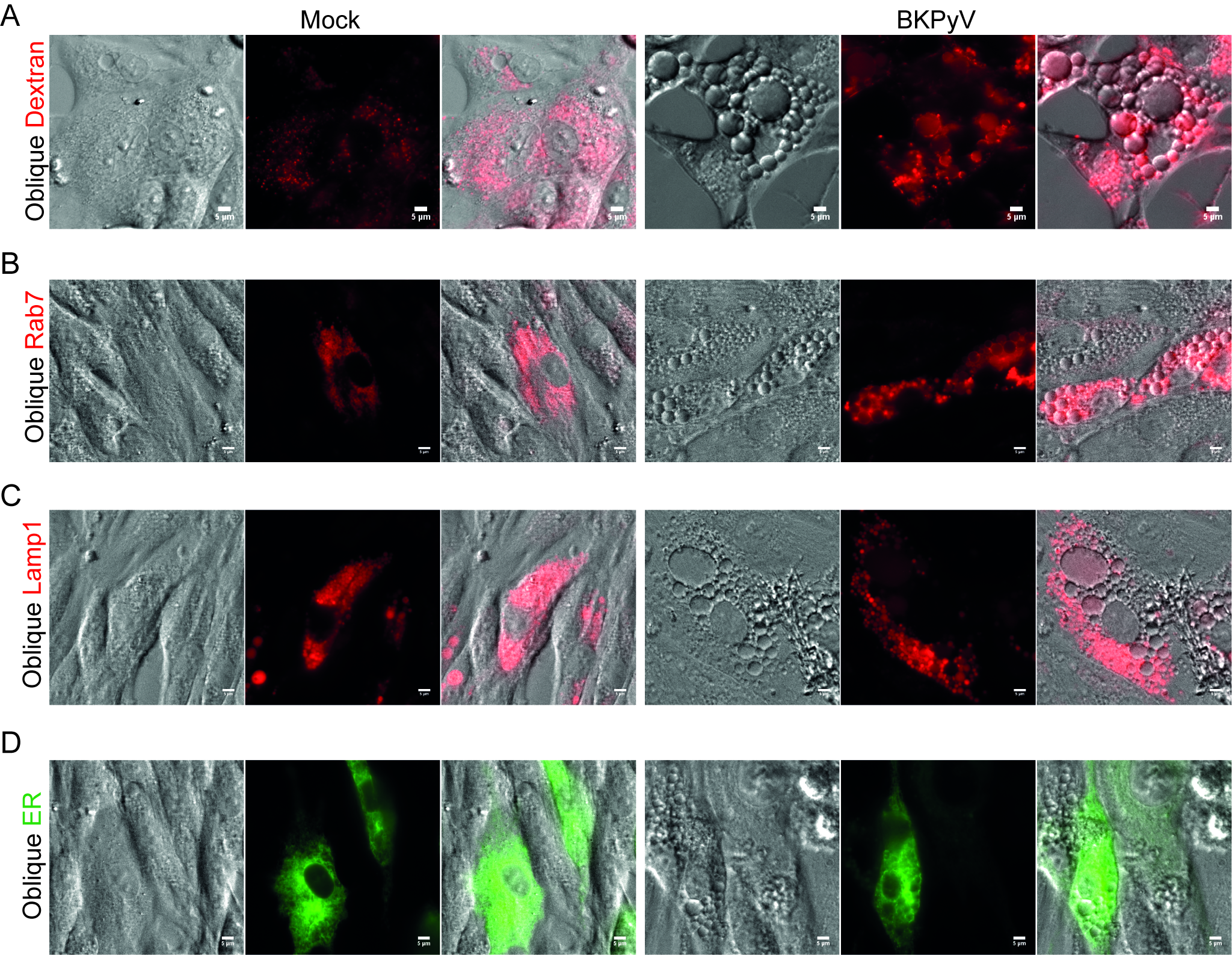

Supplement: S6 Fig — Live-cell oblique contrast and fluorescence widefield microscopy of mock-infected and BKPyV-infected RPTECs (MOI 1) with markers of endo-/lysosomes and the ER at 96 hpi. (A) RPTECs pulsed with Texas Red conjugated dextran (red) for 4 hours. (B) Transient expression of Rab7-mCherry (red). (C) Transient expression of Lamp1-mCherry (red). D) Transient expression of mTurquoise2-ER (green) and Rab7-mCherry (red). Representative images from two independent experiments are shown. Scale bar = 5 μm for all images. (TIF) [file ppat.1012681.s006.tif]

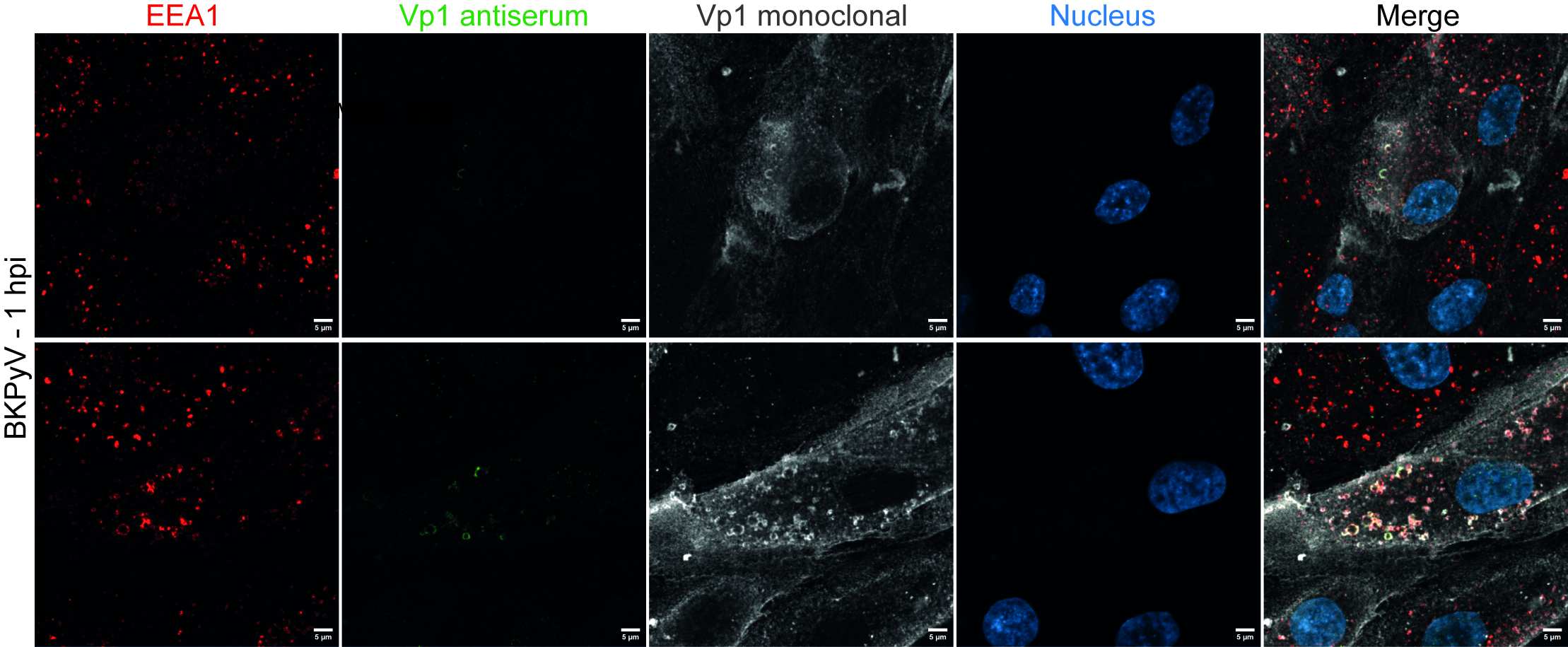

Supplement: S7 Fig — Confocal microscopy of mock-infected and BKPyV-infected RPTECs (MOI 200) at 1 hpi following immunofluorescence staining. A mouse monoclonal antibody against early endosomal protein early endosome antigen 1 (EEA1) (red) was used in combination with the rabbit BKPyV Vp1 antiserum (green) and the mouse monoclonal antibody against BKPyV Vp1 (grey). Nuclei are stained with DAPI (blue). Scale bar = 5 μm. Representative images from two independent experiments are shown. (TIF) [file ppat.1012681.s007.tif]

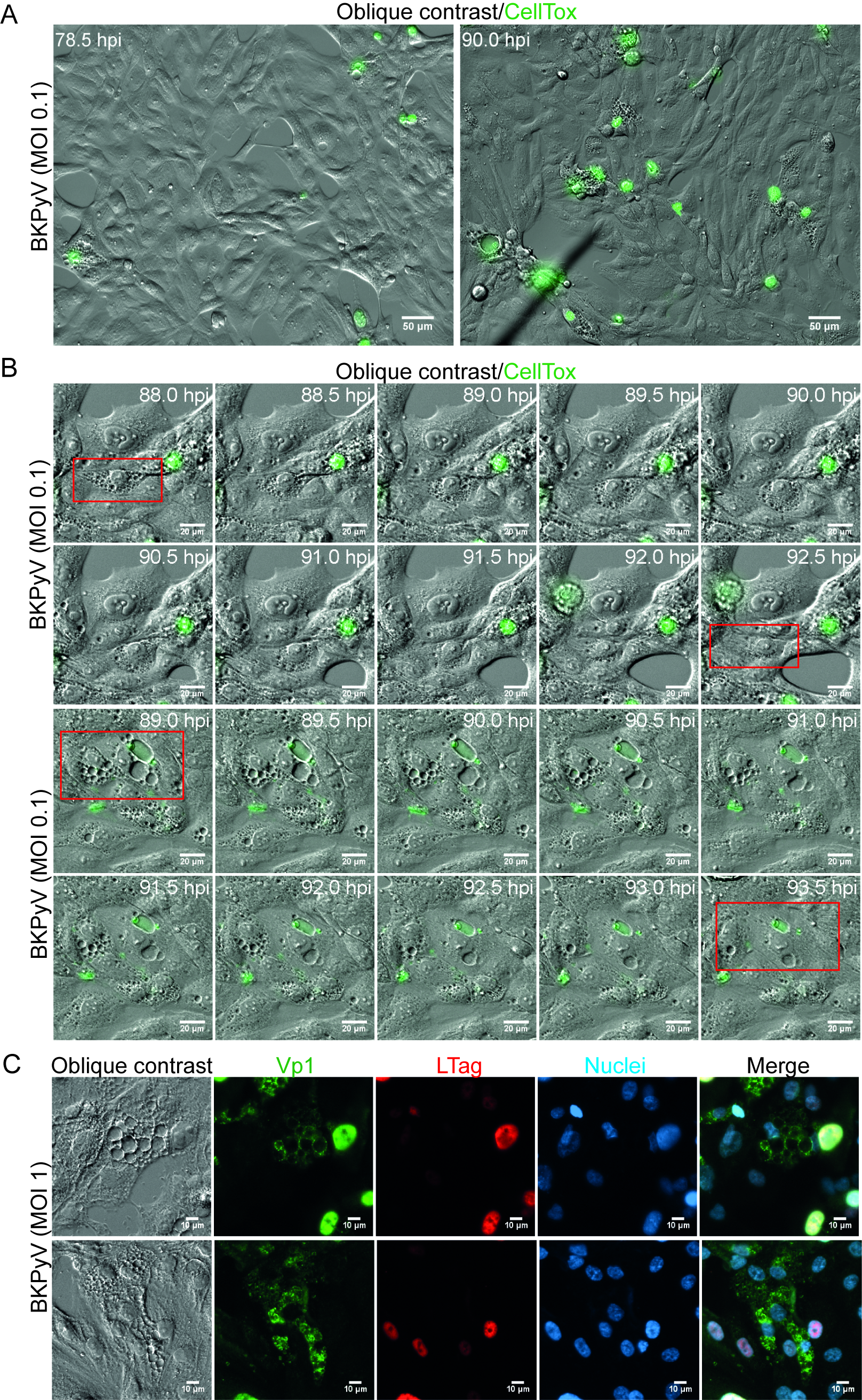

Supplement: S8 Fig — (A) Representative images from time-lapse microscopy of BKPyV-infected RPTECs (MOI 0.1) at 78.5 and 90 hpi. Cells were imaged with widefield microscopy, and dead cells were stained with CellTox (green). Scale bar = 50 μm. (B) Time-lapse microscopy of BKPyV-infected RPTECs (MOI 0.1) demonstrating two examples of transient vacuolization (red boxes). Cells were imaged with widefield microscopy, and dead cells were stained with CellTox (green). Scale bar = 20 μm. (C) Immunofluorescence staining against Vp1 (green) and LTag (red) in BKPyV-infected RPTECs (MOI 1) at 72 hpi from Fig 10B. Note RPTECs with strong cytoplasmic Vp1-staining without nuclear staining of LTag and Vp1. Nuclei were stained with DAPI. Scale bar = 10 μm. (TIF) [file ppat.1012681.s008.tif]

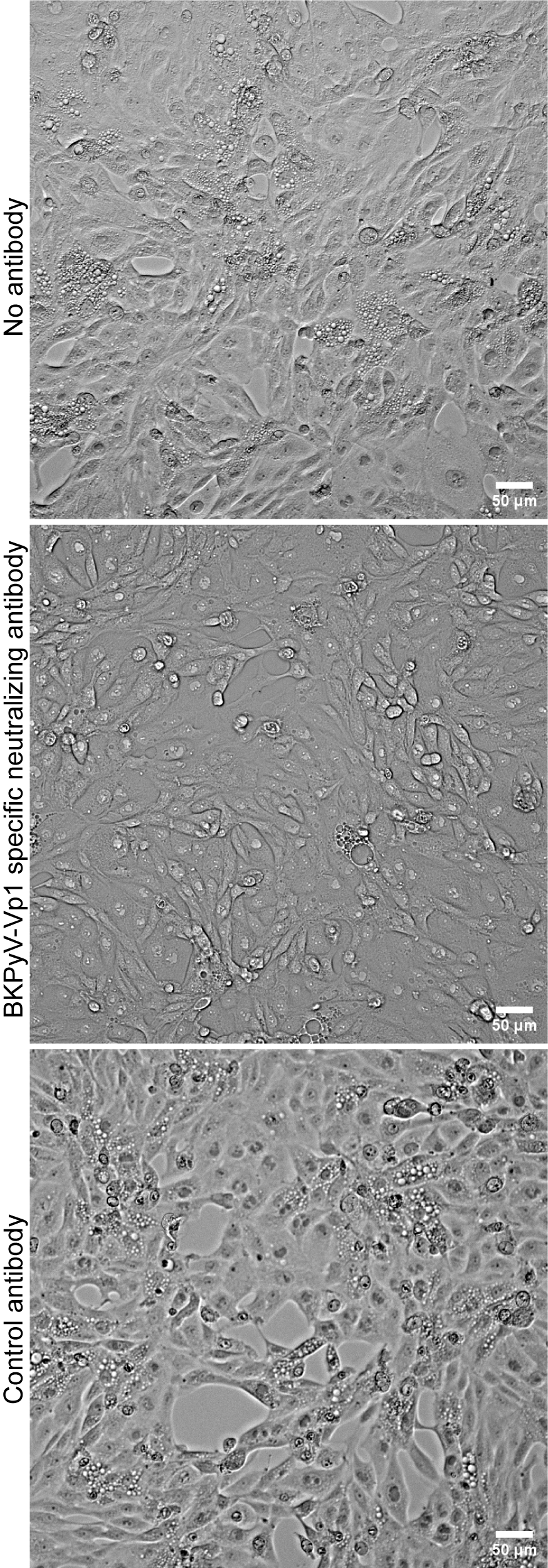

Supplement: S9 Fig — Oblique contrast microscopy of BKPyV-infected RPTECs (MOI 1) at 72 hpi. The cells were untreated or treated with either a BKPyV-Vp1 specific neutralizing antibody or a control antibody from 24 hpi. Representative images from Fig 10C and 10D are shown. Scale bar = 50 μm. (TIF) [file ppat.1012681.s009.tif]

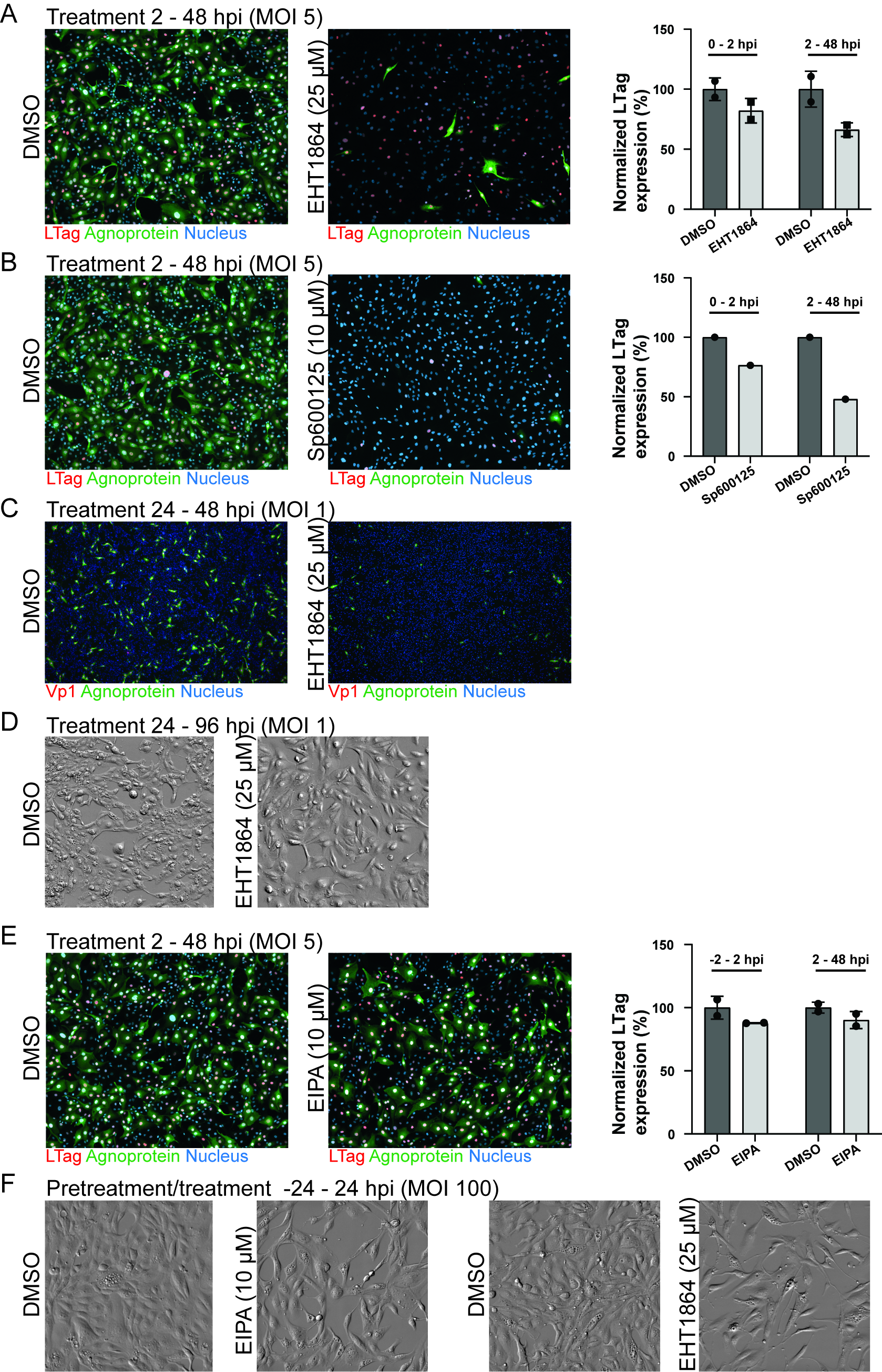

Supplement: S10 Fig — Immunofluorescence staining of LTag (red) and agnoprotein (green) in BKPyV-infected RPTECs (MOI 5) at 48 hpi after treatment 2–48 hpi with (A) EHT1864 at 25 μM and (B) Sp600125 at 10 μM. Cells with LTag expression were counted, and data was presented as normalized LTag expression (%) compared to the DMSO control. Error bars = SD and n = 2 except for Sp600125 where n = 1. (C) Immunofluorescence staining of Vp1 (red) and agnoprotein (green) in BKPyV-infected RPTECs (MOI 1) at 48 hpi after treatmentwith EHT1864 at 25 μM 24–48 hpi. Representative images from two independent experiments are shown. (D) Oblique contrast microscopy of BKPyV-infected RPTECs (MOI 1) at 96 hpi after treatment with EHT1864 at 25 μM24–96 hpi. Representative images from three individual experiments were shown. (E) Immunofluorescence staining of LTag (red) and agnoprotein (green) in BKPyV-infected RPTECs (MOI 5) at 48 hpi after treatment with EIPA at 10 μM 2–48 hpi. Cells with LTag expression were counted, and data was presented as normalized LTag expression (%) compared to the DMSO control. Error bars = SD and n = 2. (F) Oblique contrast microscopy of BKPyV-infected RPTECs (MOI 100) at 24 hpi. Cells were pretreated overnight with EHT1864 at 25 μM or EIPA at 10 μM and up to imaging at 24 hpi. Representative images from one single experiment are shown. For all experiments DMSO was used as a drug control. (TIF) [file ppat.1012681.s010.tif]

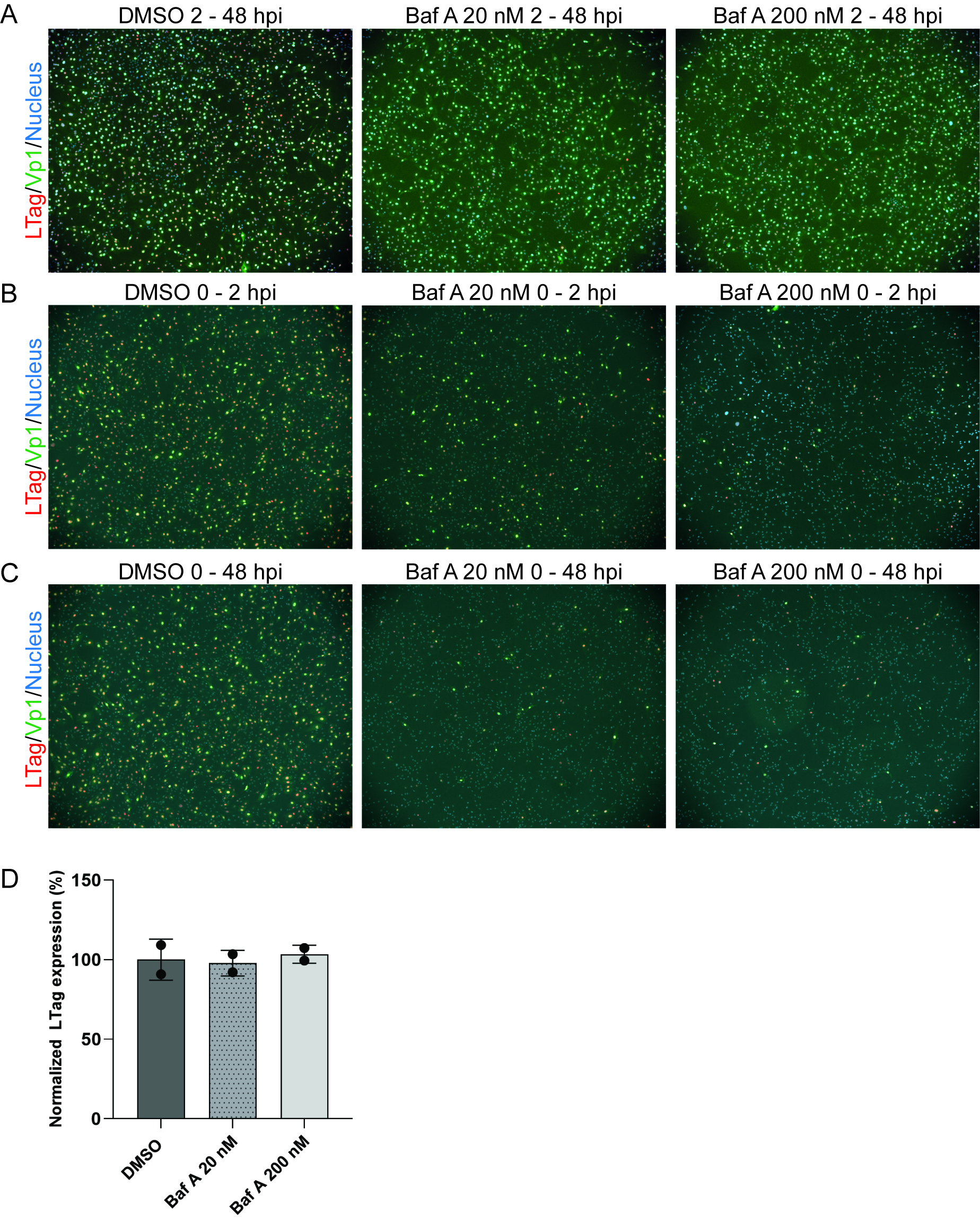

Supplement: S11 Fig — Immunofluorescence staining of DMSO or Bafilomycin A treated BKPyV (MOI 5) infected RPTECs at 48 hpi, using antibodies against LTag (red) and Vp1 (green). Cells were bafilomycin A treated from (A) 2–48 hpi, (B) 0–2 hpi, or (C) 0–48 hpi. Nuclei are stained with DAPI (blue). Representative images from two independent experiments are shown. D) Quantification of LTag expression in (A). Data is presented as LTag expression normalized to the DMSO control. Error bars represent SD and n = 2. (TIF) [file ppat.1012681.s011.tif]
