# Supplementary material for: Massive entry of BK Polyomavirus induces transient cytoplasmic vacuolization of human renal proximal tubule epithelial cells
Source: PLoS Pathog. 2024 Nov 21;20(11):e1012681. doi: 10.1371/journal.ppat.1012681 (PMC11581322; doi:10.1371/journal.ppat.1012681)
Supplement: Attachment — Submitted filename: Respose to reviewers.docx [file ppat.1012681.s019.docx]

**Responses to reviewer comments and description of changes**

***Part I - Summary
Please use this section to discuss strengths/weaknesses of study, novelty/significance, general execution and scholarship.***

***Reviewer #1: In this study, the Rinaldo group explores cytoplasmic vacuolization induced by BK polyomavirus in human renal proximal tubule epithelial cells. They show that infection at very high doses can induce rapid, transient vacuolization in both polarized and non-polarized RPTEC, whereas as low doses, vacuolization occurs late during infection, after progeny virus production and release. The vacuoles themselves appear to be enlarged endo/lysosomes, depend on virus binding to cell surface receptors, and their formation is blocked by bafilomycinA1, an inhibitor of endosomal acidification. Progeny virus release and cell lysis precede vacuole formation and do not appear dependent on it. Many of their findings parallel the SV40 system, where cell surface binding (and GM1 ganglioside engagement) triggers vacuolization, which can occur early or late upon virus replication, and binding of progeny virus induces vacuole formation late. But there are some interesting differences, including (presumably) the receptor involved, the responsible signaling pathways (at least in the RPTEC, which were not studied for SV40), the presence or absence of virions in the vacuoles, and the relative timing of vacuolization and virus release. Overall, this work appears carefully done and most of the conclusions justified (although see point three below), but the work is largely descriptive and somewhat incremental.***

We thank the reviewer for the thorough assessment of our study and the positive comments.

***Specific comments
1. The neutralizing antibody clearly inhibits vacuolization, but it might have other effects on the virus in addition to preventing receptor binding. They should be more circumspect in interpreting this experiment.***

We thank the reviewer for this comment. Unfortunately, there is no information on the epitopes used to generate the monoclonal Vp1 antibody Virostat 4942; however, we have previously shown that it can efficiently neutralize BKPyV Dunlop (serotype I) (Lorentzen et al (2023) PLOS Pathog).

In Figures 6C and D, confocal microscopy at 6 hpi demonstrates markedly reduced vacuolization and Vp1-staining in the cytoplasm when the inoculum is pre-neutralized, i.e., BKPyV was incubated with Virostat 4942 before inoculation. This shows that the antibody prevents the internalization of BKPyV. As described in a recent review, most BKPyV-specific monoclonal neutralizing antibodies interfere with the viral binding to the cellular receptor (Helle et al (2024) J Am Soc Nephrol.). To further investigate the effect of Virostat 4942, we have now performed a hemagglutination inhibition assay (HIA). Like the influenza virus, BKPyV is known to bind to the ionized sialic acids on the surface of red blood cells, bridging or agglutinating the cells. We found that pre-incubation of BKPyV with Virostat 4942 efficiently inhibits BKPyV-induced agglutination, which suggests that neutralized BKPyV is prevented from binding to the sialic acids. Although we cannot exclude that Virostat 4942 also has other effects, we find it reasonable to state that Virostat 4942 inhibits virus binding to the cellular receptor. We have included these new results and cited the paper by Helle and colleagues (**new S3A Fig and page 8, lines 152 -155).** We hope that this experiment has made the neutralizing effect of Virostat 4942 more apparent.

***2. Is the high MOI required for early vacuolization likely to be achieved in a natural infection?***

We are confident that also, during natural infections, high MOIs (MOI>10) are achieved locally around lysed cells. Even when we used a MOI as low as 0.1, late-wave vacuolization was seen with phase-contrast microscopy (Fig 1A). Furthermore, our time-lapse experiments show localized vacuolization around lysed infected cells and demonstrate in real-time that host cell lysis leads to very high local virus concentrations (Fig 9). In the result section, we have emphasized that late-wave vacuolization occurs due to a high local progeny virus concentration (**page 13, line 303 – 304),** and in the discussion part, we have now written that our results suggest the high MOIs used are physiologically relevant (**page 22, line 523 -524**).

***3. The conclusion that virus can undergo cell-to-cell spread (based on the focal clustering of cells containing late vacuoles around lysed cells) is interesting but should be better documented and justified. If cell-to-cell spread is important as stated in the abstract, late vacuole formation should be resistant to antibody neutralization, but they don’t see this in Figure 2D/E.***

We agree with the reviewer that these observations are interesting and that conventional cell-to-cell spread is typically antibody-resistant. To avoid confusion, we removed the term cell-to-cell spread from the abstract and conclusion and replaced it with local spread (**page 2, lines 40-41, and page 23, line 539)**. Moreover, we have now discussed conventional cell-to-cell spread (**page 22, lines 516 – 521)** before we, as previously, suggest that the increased local virus concentration and the following vacuolization is explained by retention of progeny virus on the surface of lysed cells. Furthermore, we have included a paragraph on kidney transplant patients. In some of these patients, a low concentration of BKPyV-specific neutralizing antibodies or neutralizing antibodies with a genotype mismatch will probably hamper BKPyV neutralization **(page 22, lines 529-531).**

***Reviewer #2: Cytoplasmic vacuolization of renal tubular cells is commonly observed in clinical histological samples, both in association with viral infections and in tubular injury of multiple other etiologies. From the morphological point of view all forms of vacuolization are lumped together.***

***The current study provides significant new information that helps understand and better categorize BKPyV associated cellular vacuolization. The transient nature of vacuolization and its relationship to the infectious cell cycle are also important findings of this study.***

***The study is comprehensive and well-illustrated.***

We thank the reviewer for the positive assessment of our experimental work.

***Reviewer #3: The manuscript from the Rinaldo group tests the idea whether BK polyomavirus, like SV40, causes vacuolisation of the cells it infects. They utilise the primary renal proximal tubular epithelial cells, which are the current best cell model for this virus and they find that BK can cause vacuole formation. They demonstrate that high tires of virus cause vacuole formation at both early and late time points, whereas lower titres only cause the phenotype at later time points, correlating with virus release. A series of analyses demonstrates both the requirements for vacuole formation and provides some information as to the cell biology of these vacuoles. The relevance of their formation in the virus lifecycle remains unclear. Overall the experiments are well performed and their are controls in the assays.***

***A major sticking point relates to the very high titres of virus used in the assays, it could call into question the physiological relevance of the findings; however, lower titres can induce vacuole formation and there are reports in the literature of vacuole formation in kidney tissues from patients with BK-induced kidney disease.***

We thank the reviewer for the positive comments and the thorough assessment of our study. Regarding the MOIs used, we agree that very high MOIs were used to induce early-wave vacuolization. Importantly, our experiments demonstrated that early- and late-wave cytoplasmic vacuolization is the same process and occurs in recently infected cells. However, it was beneficial to characterize the vacuoles early when the host cells were still intact (no cell death had occurred), and for this, a high MOI was needed. As pointed out by the reviewer, lower MOIs (MOI 0.1 - 10) did induce late-wave vacuolization. The fact that the vacuolized cells were localized directly around lysed cells demonstrates how lysis leads to a locally very high BKPyV concentration, which induced vacuolization in the surrounding cells. Therefore, we believe that the concentrations that we used are physiologically relevant. As previously mentioned under reviewer 1, item # 3, we have now written a sentence in the discussion where we claim that our results suggest that the high MOIs used are physiologically relevant (**page 22, lines 523-524).** As pointed out by the reviewer, vacuolization in kidney tissue from BKPyV nephropathy patients has been described. Our results suggest vacuolization also happens in renal tubule epithelial cells *in vivo* due to high-level replication of BKPyV.

***Part II – Major Issues: Key Experiments Required for Acceptance
Please use this section to detail the key new experiments or modifications of existing experiments that should be absolutely required to validate study conclusions.

Generally, there should be no more than 3 such required experiments or major modifications for a "Major Revision" recommendation. If more than 3 experiments are necessary to validate the study conclusions, then you are encouraged to recommend "Reject".***

***Reviewer #1: Item #3 above should be address substantively***

See above, under Reviewer 1, item #3.

***Reviewer #2: The experimental aspects are well planned and well executed.***

We thank the reviewer for the positive comments.

***Reviewer #3: The authors could further test if this is truly dependent on GT1b/GD1b by either enzymatic inhibition of their production or siRNA/shRNA knockdown and performing the infection assays.***

We thank the reviewer for this helpful suggestion. Since RPTECs are primary cells, they are not so easy to transfect. Moreover, we only keep them in culture for about one week, making knockdown or long-term experiments difficult. Instead, we have now assessed the effect of neuraminidase treatment on vacuolization. Neuraminidase is an enzyme that catalyzes the cleavage of sialic acids from a wide variety of glycoproteins, glycolipids, and oligosaccharides. As BKPyV uses sialic acids for attachment to the host cells, neuraminidase treatment inhibits BKPyV replication (Sinibaldi et al (1990). Arch Virol.). As expected, pre-treatment of RPTECs with neuraminidase significantly reduced vacuolization, demonstrating that virus binding to sialic acids is necessary for vacuolization (**new Fig. 2F and page 8, lines 169-173**). Although we did not directly investigate the dependents of GT1b/GD1b for vacuolization, we noted that CV-1 and HeLa cells that appear to have less GT1b/GD1b than RPTECs, take up BKPyV but do not form vacuoles, supporting a dependence on GT1b/GD1b (**new S2C Fig, page 7, lines 135-139 and page 19, lines 426-436).

*The authors could further demonstrate that replication is not needed by using VLPs in addition to the heat inactivated virus.***

We thank the reviewer for this suggestion. Vacuoles are formed before 6 hpi when virions are still lining the inner vacuole surface (Fig 6C and Fig 7), which argues that BKPyV replication is not needed for vacuole formation. To further strengthen our evidence that replication is unnecessary for early-wave vacuolization, we have now utilized BKVLPs. Infection of RPTECs with a dose of BKVLPs equivalent to BKPyV MOI 200 (based on the hemagglutination titer) caused massive vacuolization. This demonstrates that replication is unnecessary for early-wave vacuolization (**new Fig 2G and pages 8-9, lines 173-176).** Moreover, we did an experiment where we mixed different amounts of VLPs and BKPyV, and this confirmed that late wave-vacuolization is dependent on BKPyV replication and progeny release and does, therefore, not occur in cells given only BKVLPs (**new S4 Fig and page 9, lines 176-182**).

**Part III – Minor Issues: Editorial and Data Presentation Modifications**
Please use this section for editorial suggestions as well as relatively minor modifications of existing data that would enhance clarity.

***Reviewer #1: 1. On line 152, they should emphasize that they used a low MOI to explore late vacuole formation dependent on virus replication and progeny virus production.***

We thank the reviewer for this suggestion. We have added the following sentence: “Here, we used a MOI of 1, which was not enough virus to induce early-wave vacuolization but yielded subsequent late-wave vacuolization.” **(page 8, lines 163 – 165)**.

***Reviewer #2: The authors demonstrated that "early" and "late" vacuolization represent an identical process related to the massive entry of BKPYV. I suggest that further qualification be used after early and late vacuolization (such as early or late wave of vacuolization, or early and late surge of vacuolization) indicating that the temporality is related to the time in the course of the study, rather than to the individual cell infectious cycle. In the manuscript the idea eventually becomes clear, however, until the results for the findings in the late vacuolization are presented, there is potential ambiguity regarding this issue.***

We thank the reviewer for the suggestion and have changed the definition to early-wave and late-wave vacuolization as suggested **(page 6, lines 117-120)**. Moreover, for increased clarity, we have included the following sentence in the result part: “Notably, the temporal aspect, early and late, is related to the time of infection or inoculation of the cell culture and not the replication cycle of the individual cells in the cell culture.” **(page 6, lines 120-122).** Moreover, the following sentence was included in the discussion: “Notably, early- and late-wave vacuolization are identical processes induced by binding and uptake of BKPyV from the initial inoculum or released progeny virus, respectively, and consequently occur in cells that have been recently infected.” **(page 18, lines 407-410).**

***Reviewer #3: The discussion needs to provide clearer thoughts on how BK induces vacuoles as presumably the virus induces productive infection in HeLa and CV1 cells but does not cause vacuole formation, hence the infection process per se does not seem sufficient for this (yet that is the suggestion in the RPTE cells). What is different between the cell types?***

We thank the reviewer for this suggestion. Our neutralization and neuraminidase experiments show that BKPyV binding to sialic acids is necessary for viral entry and vacuolization. To better understand the lack of vacuolization in CV-1 and HeLa cells, we have now compared the binding and uptake of BKPyV in CV-1, Hela cells, and RPTECs. The cells were infected, and immunofluorescence staining for Vp1 and confocal microscopy was performed at 6 hpi. Interestingly, this revealed similar levels of Vp1 in these cells, indicating that the binding and uptake was similar (**new Fig S2C and page 7, lines 135 – 139**). The result is puzzling. Based on previous studies (Bennett et al (2013) J Virol, Luo et al (2016) mBio), Moriyama et al (2008) Virology, and Markwell et al (1984) Biochimica et Biophysica Acta (BBA) - Biomembranes), we speculate that this is due to la combination of less GT1b and GD1b on the plasma membrane of CV-1 and HeLa cells than on RPTECs and a different cell-type specific transport (**page 19, lines 426-436).** However, investigating the detailed entry and intracellular transport of BKPyV in CV-1 and HeLa cells is outside the focus of this manuscript.

***It would be very useful for the authors either in the methods or in the results to provide clearer explanation as to why different MOIs are used for different experiments (these can vary quite significantly between experiments in each figure).***

We thank the reviewer for this suggestion. For increased clarity, we have provided a more in-depth explanation of the choice of MOI in the Material and Methods section (**page 24, lines 556 – 562).**
